# Multiple behaviour change intervention and outcomes in recently diagnosed type 2 diabetes: the ADDITION-Plus randomised controlled trial

**DOI:** 10.1007/s00125-014-3236-6

**Published:** 2014-04-24

**Authors:** Simon J. Griffin, Rebecca K. Simmons, A. Toby Prevost, Kate M. Williams, Wendy Hardeman, Stephen Sutton, Søren Brage, Ulf Ekelund, Richard A. Parker, Nicholas J. Wareham, Ann Louise Kinmonth, on behalf of the ADDITION-Plus study team

**Affiliations:** 1 MRC Epidemiology Unit, University of Cambridge School of Clinical Medicine, Cambridge, UK; 2 The Primary Care Unit, Department of Public Health and Primary Care, University of Cambridge, Robinson Way, Cambridge, CB2 0SR UK; 3 Department of Primary Care and Public Health Sciences, King’s College London, London, UK; 4 Department of Sports Medicine, Norwegian School of Sport Sciences, Oslo, Norway

**Keywords:** ADDITION-Plus, Diabetes, General practice, Health behaviour, Randomised trial

## Abstract

**Aims/hypothesis:**

The aim of this study was to assess whether or not a theory-based behaviour change intervention delivered by trained and quality-assured lifestyle facilitators can achieve and maintain improvements in physical activity, dietary change, medication adherence and smoking cessation in people with recently diagnosed type 2 diabetes.

**Methods:**

An explanatory randomised controlled trial was conducted in 34 general practices in Eastern England (Anglo–Danish–Dutch Study of Intensive Treatment in People with Screen Detected Diabetes in Primary Care-Plus [ADDITION-Plus]). In all, 478 patients meeting eligibility criteria (age 40 to 69 years with recently diagnosed screen or clinically detected diabetes) were individually randomised to receive either intensive treatment (*n* = 239) or intensive treatment plus a theory-based behaviour change intervention led by a facilitator external to the general practice team (*n* = 239). Randomisation was central and independent using a partial minimisation procedure to balance stratifiers between treatment arms. Facilitators taught patients skills to facilitate change in and maintenance of key health behaviours, including goal setting, self-monitoring and building habits. Primary outcomes included physical activity energy expenditure (individually calibrated heart rate monitoring and movement sensing), change in objectively measured fruit and vegetable intake (plasma vitamin C), medication adherence (plasma drug levels) and smoking status (plasma cotinine levels) at 1 year. Measurements, data entry and laboratory analysis were conducted with staff unaware of participants’ study group allocation.

**Results:**

Of 475 participants still alive, 444 (93%; intervention group 95%, comparison group 92%) attended 1-year follow-up. There were no significant differences between groups in physical activity (difference: +1.50 kJ kg^−1^ day^−1^; 95% CI −1.74, 4.74), plasma vitamin C (difference: −3.84 μmol/l; 95% CI −8.07, 0.38), smoking (OR 1.37; 95% CI 0.77, 2.43) and plasma drug levels (difference in metformin levels: −119.5 μmol/l; 95% CI −335.0, 95.9). Cardiovascular risk factors and self-reported behaviour improved in both groups with no significant differences between groups.

**Conclusions/interpretation:**

For patients with recently diagnosed type 2 diabetes receiving intensive treatment in UK primary care, a facilitator-led individually tailored behaviour change intervention did not improve objectively measured health behaviours or cardiovascular risk factors over 1 year.

**Trial registration:**

ISRCTN99175498

**Funding:**

The trial is supported by the Medical Research Council, the Wellcome Trust, National Health Service R&D support funding (including the Primary Care Research and Diabetes Research Networks) and National Institute of Health Research under its Programme Grants for Applied Research scheme. The Primary Care Unit is supported by NIHR Research funds. Bio-Rad provided equipment for HbA_1c_ testing during the screening phase.

**Electronic supplementary material:**

The online version of this article (doi:10.1007/s00125-014-3236-6) contains peer-reviewed but unedited supplementary material, which is available to authorised users.

## Introduction

Well-organised care, including regular recall and review of patients, prompting of doctors, feedback on goal attainment, and continuing medical education and guidelines, are associated with reductions in risk factors among people with type 2 diabetes [1, 2]. In addition, intensive pharmacological treatment of risk factors can reduce cardiovascular morbidity and mortality [3, 4]. However, these benefits depend on people with diabetes taking their medication as prescribed, eating a healthy diet, being physically active on a regular basis and avoiding smoking.

There is some evidence that patient education, training patients in self-management and using interventions incorporating well-specified behaviour change techniques can be effective, at least in the short term [5]. However, only a minority of people with type 2 diabetes in the UK have attended a structured education programme to assist with behaviour change, and where attendance has occurred the effectiveness of the programme in practice is largely uncertain [6].

The Early Activity In Diabetes (Early ACTID: ISRCTN92162869) and Action for Health Diabetes (Look-AHEAD: NCT00017953) trials have demonstrated beneficial effects on cardiovascular risk factors of adding intensive lifestyle interventions to the primary care of diabetes patients [7, 8]. These studies were selective in their behavioural focus. Many previous studies have been conducted in research clinics or specialist settings and have not clearly characterised the comparison condition. Behavioural interventions and their hypothesised mechanisms of action are rarely clearly specified and the delivery of the intervention is not often assessed. Most trials have relied on self-reported measures of behaviour, which are imprecise and subject to recall bias, and do not assess adherence to medication. This limits evaluation of the effects of behavioural interventions independent of organisational and pharmacological components, and cannot inform subsequent integration of the most effective components into routine practice.

We aimed to address these uncertainties in the Anglo–Danish–Dutch Study of Intensive Treatment in People with Screen Detected Diabetes in Primary Care-Plus (ADDITION-Plus) trial by evaluating the effect of a theory-based behaviour change intervention delivered by trained and quality-assured lifestyle facilitators, external to the primary care team, on objectively measured health behaviours (physical activity, diet change, medication adherence and smoking) among people with recently diagnosed type 2 diabetes receiving intensive general practice care [9].

## Methods

### Study design

**Fig. 1:**
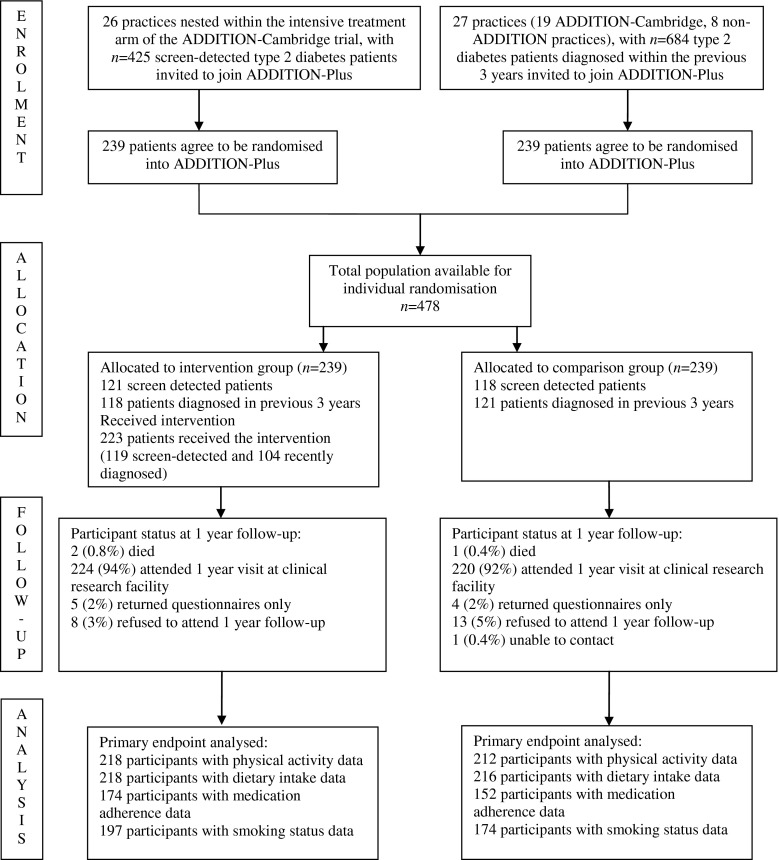
Study design and participant flow in the ADDITION-Plus trial

The study design and rationale have been reported previously (2002–2007) [9]. Patients were recruited from 34 general practices in the East of England. The majority of practices (*n* = 26) were participating in the intensive treatment arm of the ADDITION-Cambridge trial of treatment of people with diabetes detected by screening (ISRCTN86769081) [10]. A further eight practices were recruited to increase the participation of recently clinically diagnosed patients (Fig. 1). Eligibility criteria were initially assessed by general practice staff and included age 40–69 years with type 2 diabetes following screening in the ADDITION study or clinical diagnosis during the previous 3 years. Exclusion criteria included patients who had a psychotic illness or an illness with a likely prognosis of <1 year and women who were pregnant or lactating. Of 425 eligible screen-detected patients and 684 patients clinically diagnosed within the previous 3 years, 239 from each group agreed to be individually randomised to receive intensive treatment alone (comparison group) or in conjunction with a facilitator-led behaviour change intervention (intervention group).

### Randomisation

Randomisation was central using a partial minimisation procedure to balance stratifiers (age, sex and general practice; and within screen-detected and clinically diagnosed subgroups: BMI, self-reported smoking and medication adherence [11]) between the arms [9]. Randomisation was undertaken independently of study coordination or knowledge of or contact with participants or their data, other than the stratifiers. Ethical approval was obtained from the Eastern MultiCentre Research Ethics Committee (reference no. 02/5/54). All participants provided written informed consent.

### Intervention

#### Comparison group: intensive treatment

A number of features were added to routine multidisciplinary primary care of diabetes to achieve intensive treatment in both trial groups as previously described [9, 10] (see Electronic Supplementary Material [ESM] for further details).

#### Intervention group: intensive treatment plus facilitator-led behaviour change intervention

Participants in the intervention group received intensive treatment (described above) plus a facilitator-led, individually tailored behaviour change intervention, based on psychological theory and evidence. Full details of the intervention have been described previously (see ESM) [9]. In brief, the intervention was delivered by three female trained lifestyle facilitators, who were not part of the general practice team. The facilitators used detailed protocols to guide each contact with the participant and received ongoing supervision and feedback from a clinical psychologist. The behaviours targeted in the intervention were physical activity, dietary intake, medication adherence and smoking cessation. Facilitators taught patients a range of self-regulatory skills to achieve behaviour change and maintenance over time, supported by a manual describing the skills. The intervention was delivered over 1 year at the participants’ surgeries and included a 1 h introductory meeting followed by six 30 min meetings and four brief phone calls.

### Measurements and endpoints

Baseline and 1 year measurements were undertaken at outpatient clinical research facilities by trained staff following standard operating procedures. Double data entry of anthropometric and questionnaire measures was undertaken by independent agencies. Measurements, data entry and laboratory analysis were conducted with staff unaware of participants’ study group allocation.

### Primary outcomes

#### Objectively measured health behaviours

Physical activity was assessed at 1 year using a combined heart rate and movement sensor (Actiheart, CamNtech, Cambridge, UK), which participants were advised to wear continuously for at least 4 days [12]. A graded treadmill walk test was used to individually calibrate heart rate [13] and to estimate cardiorespiratory fitness by extrapolation of the heart rate/oxygen consumption relationship to age-predicted maximal heart rate [14]. Time-series data were pre-processed [15] and summarised into physical activity energy expenditure (PAEE, kJ kg^−1^ day^−1^) [16]. Data from participants without a treadmill test (*n* = 79) were processed using an adjusted group calibration equation based on treadmill tests.

Intake of fruit and vegetables was assessed at baseline and 1 year by measurement of plasma vitamin C levels using a Fluoroskan Ascent FL fluorometer (Fisher Scientific UK, Loughborough, UK) [17]. Smoking status was assessed at 1 year by analysis of plasma cotinine levels using an Immulite Nicotine metabolite solid phase competitive chemiluminescent immunoassay (Siemens Healthcare Diagnostics, Llanberis, UK) and self-report. Self-reported non-smokers with cotinine levels exceeding ≥15 ng/ml (*n* = 12) were re-classified as smokers [18]. Participants were advised to take their medication as usual on the day of testing. Medication adherence was assessed at 1 year follow-up by measurement of plasma concentrations of the three drugs that provided maximum participant coverage (metformin, simvastatin and atorvastatin) using liquid-chromatography–mass-spectrometry after protein precipitation extraction [9].

### Secondary outcomes

#### Self-reported health behaviours

Physical activity, dietary intake and adherence to hypoglycaemic and other medication were assessed using the previously validated EPIC–Norfolk Physical Activity Questionnaire [19], Food Frequency Questionnaire [20] and the Medication Adherence Report Schedule (MARS) [11], respectively. At 1 year, participants were asked whether or not they had made changes to their physical activity and dietary behaviours in the preceding 12 months.

#### Modelled risk of cardiovascular disease

This was calculated for individuals without a prior cardiovascular disease (CVD) event using the UK Prospective Diabetes Study (UKPDS) model [21].

#### Biochemical and clinical measures

Biochemical measures were assessed using standard assays (ESM) [9]. Detailed measurement protocols for blood pressure, height, weight, waist circumference and body fat percentage have been described previously [9]. Angina was assessed using the Rose angina questionnaire [22]. Neuropathy was evaluated using an adapted version of the Michigan Neuropathy Screening Instrument [23].

#### Functional status, healthy utility, anxiety, well-being, quality of life, treatment satisfaction and relational empathy

The following generic and disease-specific instruments were used: Short-Form 36 (SF-36) [24], EuroQol (EQ-5D) [25], the short form of the state scale of the Spielberger State-Trait Anxiety Inventory [26], diabetes Well-Being Questionnaire [27], Audit of Diabetes Dependent Quality of Life [27] and Diabetes Treatment Satisfaction Questionnaire [27]. All patients completed the Consultation and Relational Empathy (CARE) measure in relation to the general practitioner and practice nurse [28], and a question concerning satisfaction with diabetes services. In addition, participants in the intervention group completed the CARE measure in relation to the lifestyle facilitator.

#### Beliefs about behaviour change, illness perceptions and habit

A questionnaire developed according to the theory of planned behaviour (TPB) model [29, 30] assessed selected cognitions about becoming more physically active, eating a lower-fat diet, taking medication and smoking cessation: intention, perceived behavioural control and behavioural beliefs. Full details are reported elsewhere [9]. Participants also completed the consequences and treatment control subscales (11 items) of the Illness Perception Questionnaire-Revised [31, 32] and a nine-item closed-response questionnaire covering basic knowledge of diabetes and its management [33]. At 1 year, participants were asked to write down the most important change they had made in their physical activity and dietary intake [34]. Cronbach’s alpha for TPB measures and the Illness Perception Questionnaire measures demonstrated high internal consistency. However, internal consistency was low (0.55) for diabetes treatment control and perceived behavioural control in relation to stopping smoking.

### Statistical analysis

We planned to recruit 500 participants. Follow-up of 400 individuals would provide 80% power at the 5% level of significance to detect between-arm differences of 0.017 kJ kg^−1^ min^−1^ in PAEE (anticipating a mean [SD] of 0.078 [0.058] kJ kg^−1^ min^−1^) [35], 4.0 μmol/l in change from baseline in plasma vitamin C adjusted further for baseline (based on a mean [SD] of 53 [19] μmol/l and test–retest correlation of 0.67 [36]) and 9.5% in smoking prevalence (control prevalence 17.9%).

The effectiveness of the intervention on primary outcomes was assessed using between-arm intention-to-treat analysis, using linear and logistic regression for continuous and binary outcomes, respectively. Continuous outcomes adjusted for baseline where measured. Participants with missing baseline values were retained in the analysis by using the missing indicator method [37]. Effect sizes were reported with 95% CIs. Statistical significance was set at *p* < 0.05. Continuous outcomes indicating non-normality or skewness (simvastatin, atorvastatin, triacylglycerol, alanine aminotransferase and UKPDS risk score) were natural log-transformed before analysis and their CIs were shown on the log-scale. Where non-normality could not be resolved by log-transformation, 95% CIs were estimated using semi-parametric bias corrected and accelerated bootstrap regression with 1,999 resamples. We conducted a per protocol analysis of each primary outcome, in which the per protocol population comprised comparison-arm participants and those intervention-arm participants who attended the introductory and initial three core-intervention sessions. Sensitivity analysis was undertaken to assess the impact of excluding participants with missing primary outcome data. Rubin’s multiple imputation method with 30 multiple imputation datasets [38] was used together with two contrasting scenarios for the intervention effect in excluded participants. The first optimistically assumed that excluded participants achieved the same full intervention effect as observed in participants with the outcome data, and the second pessimistically assumed that no intervention effect was achieved. Subgroup analyses were confined to the comparison of intervention effects on primary outcomes by mode of diagnosis (screen-detected or recently diagnosed).

## Results

Figure 1 shows the trial profile. Participating practices were largely comparable with the average English practice in terms of list size, diabetes prevalence and general practice/nurse whole-time equivalents (data taken from the National Primary Care Database; www.population-health.manchester.ac.uk/primarycare/npcrdc-archive/archive/ProjectDetail.cfm/ID/10.htm). However, the median (interquartile range) Index of Multiple Deprivation score for ADDITION-Plus practices (11.7 [6.6–15.5]) suggested that they served less deprived communities than the average English practice (21.2 [12.2–36.1]) (data taken from the UK National Primary Care Database). Four hundred and seventy-eight eligible participants were recruited to the study (intervention group *n* = 239; comparison group *n* = 239) and attended baseline measurement. Baseline characteristics were similar in the two trial groups (Table 1). The majority of ADDITION-Plus participants were white men with a mean age of 60 years. In all, 51% of participants were in full- or part-time employment, and 62% had attended full-time education after the age of 16 years. There was one death in the comparison group and two deaths in the intervention group within 13 months of recruitment. Of those still alive, 444/475 (93%; intervention group: 95%, comparison group: 92%) returned for follow-up health assessment after a mean of 1.1 years (SD 0.2). There was no significant difference in baseline characteristics between those who attended follow-up health assessment and those who did not (data not shown).

**Table 1:** Baseline characteristics of ADDITION-Plus trial participants (*n* = 478)

Participant characteristics	Comparison group (*n* = 239)	Intervention group (*n* = 239)
Mean age (SD), years	59.8 (7.5)	59.5 (7.5)
Men	148 (61.9)	150 (62.8)
White ethnicity	234 (97.9)	232 (97.1)
In full- or part-time employment	122/238 (51.3)	123/239 (51.5)
Full-time education after 16 years of age	145/236 (61.4)	146/236 (61.9)

Values are *n* (%) unless specified

### Objectively measured health behaviours

Table 2 shows the outcomes of objectively measured health behaviours. PAEE levels were similar in both groups at 1 year. We observed small increases in plasma vitamin C levels in both groups over 12 months, with levels approximating national trends (National Diet and Nutrition Survey 2002 [39]) and not significantly differing by group. The proportion of smokers was similar in the intervention and comparison groups at 1 year, reflecting national smoking rates [40]. There were no differences between groups in the proportion of patients prescribed metformin, simvastatin and atorvastatin (data not shown), the mean prescribed total daily dose of each drug (data not shown) and the plasma drug levels.

**Table 2:** Objectively measured health behaviours by study group and between-group differences at 1 year in the ADDITION-Plus trial

Health behaviours	Comparison group	Intervention group	Difference between groups (95% CI)	*p* value
PAEE, kJ kg^−1^ day^−1a^	33.7 (15.9) (*N* = 212)	35.2 (18.2) (*N* = 218)	+1.50 (−1.74, 4.74)	0.36
Plasma vitamin C, μmol/l	56.2 (25.1) (*N* = 216)	53.4 (25.0) (*N* = 218)	−3.84 (−8.07, 0.38)^b^	0.07
Smoking, %; cotinine validated (*n*/*N*)	13.2 (23/174)	17.3 (34/197)	1.37^c^ (0.77, 2.43)	0.28
Drug plasma level, μmol/l				
Metformin	1,310.0 (741.7) (*N* = 82)	1,190.5 (725.6) (*N* = 100)	−119.5 (−335.0, 95.9)	0.28
Log simvastatin^d^	−1.07 (1.73) (*N* = 30)	−1.43 (1.45) (*N* = 42)	−0.36 (−1.10, 0.39)	0.35
Log atorvastatin^d^	1.07 (1.01) (*N* = 40)	0.91 (1.02) (*N* = 32)	−0.16 (−0.63, 0.32)	0.52

Values are mean SD unless otherwise stated; *N* is the number with data available

^a^Physical activity is individually calibrated where available (*n* = 351), otherwise group calibrated (*n* = 79)

^b^Difference between groups is adjusted for baseline values

^c^Results are expressed as ORs

^d^Results are expressed on the natural log-scale since original variable showed substantial skewness

### Self-reported health behaviours

Participants reported increases in levels of total physical activity and consumption of fruit, vegetables and fibre, and reduction in consumption of fat and alcohol over 1 year, with no significant difference between groups (Table 3). There was little change in the proportion smoking with no significant between-group differences. Self-reported adherence to general medication increased in both groups from baseline to 1 year follow-up. Medication adherence for both general and diabetes medication was high in both groups at 1 year (defined as a score of >23/25 on the MARS questionnaire), with no significant difference between groups. A significantly higher number of patients in the intervention than in the comparison group reported that they had made positive changes to their diet and physical activity in the preceding 12 months.

**Table 3:** Baseline and 1 year follow-up self-reported behaviours by study group and between-group differences in the ADDITION-Plus trial

Self-reported behaviour	Comparison group		Intervention group		Adjusted difference between groups (95% CI)
	Baseline	1 year	Baseline	1 year	
Physical activity					
Total physical activity, MET h/week	78.6 (48.0)	80.1 (49.5)	90.0 (55.1)	95.2 (55.7)	+7.51 (−0.009, 15.0)
Recreational activity, h/week	9.63 (9.81)	10.4 (10.6)	12.0 (13.0)	13.1 (12.9)	+1.46 (−0.40, 3.34)^a^
Vigorous activity, h/week	1.03 (2.41)	1.04 (2.81)	1.33 (3.59)	1.42 (3.39)	+0.23 (−0.32, 0.72)^a^
Time spent in sedentary activity, h/week	38.7 (19.6)	36.4 (17.7)	35.3 (15.5)	33.4 (16.3)	−0.60 (−2.57, 1.38)
Television viewing, h/week	24.4 (12.8)	23.9 (11.7)	24.0 (10.2)	23.4 (10.3)	−0.20 (−1.49, 1.10)
Proportion reporting change in physical activity at 1 year, %	–	41.0 (91/222)	–	74.9 (170/227)	4.29 (2.87, 6.42)^b^
Diet					
Total fat, g/day	68.7 (30.3)	60.3 (23.3)	68.3 (29.6)	60.6 (22.2)	+0.41 (−3.11, 3.94)
Fat as percentage of energy, %	32.0 (6.2)	31.2 (6.0)	31.3 (5.7)	30.4 (5.9)	−0.45 (−1.41, 0.51)
Polyunsaturated:saturated fat ratio	0.58 (0.23)	0.67 (0.27)	0.62 (0.28)	0.68 (0.26)	−0.01 (−0.06, 0.03)
Fibre, g/day	18.6 (7.3)	19.0 (7.3)	18.6 (7.2)	19.8 (7.0)	0.82 (−0.31, 1.95)
Plasma vitamin C, mg/day	147.4 (71.2)	145.5 (66.0)	141.0 (72.2)	146.2 (66.5)	3.89 (−6.62, 14.40)
Total energy, kJ	7,966 (2,628)	7,200 (2,092)	8,099 (2,757)	7,446 (2,110)	188.0 (−136.5, 512.5)
Fruit food group (11 items), g/day	277 (197)	312 (222)	266 (198)	312 (212)	4.89 (−31.2, 41.2)^a^
Vegetable food group (19 items), g/day	237 (128)	246 (148)	231 (161)	247 (135)	4.76 (−19.0, 26.4)^a^
Alcohol, units/week	8.5 (14.3)	7.3 (11.1)	9.5 (13.6)	7.8 (10.6)	−0.25 (−1.45, 1.24)^a^
Proportion reporting change in diet at 1 year, %	–	57.2 (127/222)	–	77.4 (175/226)	2.57 (1.70, 3.87)^b^
Smoking					
Self-reported smokers, %	14.0 (31/222)	11.7 (26/222)	15.0 (34/227)	15.0 (34/227)	2.63 (0.76, 9.05)^b^
Medication adherence					
Adhering to hypoglycaemic medication, %^c^	–	81.2 (108/133)	–	85.7 (132/154)	1.39 (0.74, 2.60)^b^
Adhering to general medication, %^c^	73.1 (147/201)	75.6 (161/213)	69.5 (148/213)	78.4 (174/222)	1.25 (0.79, 1.99)^b^

Values are mean (SD) unless stated

^a^Model residuals showed non-normality, which could not be resolved after log-transformation. Therefore, 95% CIs were calculated using the semi-parametric bias corrected and accelerated bootstrap regression method (with 1,999 resamples)

^b^Results are expressed as ORs

^c^Adherence defined as a score of >23/25 on the MARS questionnaire

### Clinical and biochemical measures

Table 4 shows the baseline and follow-up anthropometric, clinical and biochemical measures. We observed small reductions in cardiovascular risk factors in both groups over 1 year. The majority of between-group differences favoured the intervention group but none achieved statistical significance.

**Table 4:** Baseline and follow-up anthropometric, clinical and biochemical measures by study group and between-group differences in the ADDITION-Plus trial

Measure	Comparison group		Intervention group		Adjusted difference between groups (95% CI)
	Baseline	1 year	Baseline	1 year	
BMI, kg/m^2^	32.8 (5.7)	32.3 (5.7)	32.7 (5.3)	32.1 (5.2)	−0.11 (−0.44, 0.22)
Waist circumference, cm	110.7 (15.1)	109.5 (15.2)	110.9 (12.4)	109.1 (11.8)	−0.63 (−1.70, 0.44)
Body fat, %	42.1 (11.9)	41.8 (11.2)	42.8 (12.3)	42.0 (11.8)	−0.30 (−1.72, 1.12)
Systolic BP, mmHg	134.4 (19.3)	128.3 (17.4)	138.2 (19.1)	132.1 (17.4)	1.76 (−0.92, 4.43)
Diastolic BP, mmHg	79.1 (10.9)	75.1 (9.8)	81.6 (10.0)	77.7 (8.8)	1.43 (−0.07, 2.92)
Cardiorespiratory fitness, ml/kg/min	–	30.9 (9.5)	–	30.3 (8.6)	−0.61 (−2.63, 1.42)
HbA_1c,_ %	7.01 (1.23)	6.67 (0.95)	7.23 (1.62)	6.66 (0.94)	−0.09 (−0.25, 0.07)
HbA_1c_, mmol/mol	53.1	49.4	55.5	49.3	
Total cholesterol, mmol/l	4.90 (1.16)	4.31 (0.86)	4.96 (0.98)	4.33 (0.91)	−0.005 (−0.16, 0.15)
HDL-cholesterol, mmol/l	1.20 (0.35)	1.19 (0.32)	1.17 (0.35)	1.19 (0.31)	0.02 (−0.02, 0.05)
LDL-cholesterol, mmol/l	2.87 (0.99)	2.34 (0.76)	2.89 (0.91)	2.29 (0.77)	−0.06 (−0.19, 0.08)
Log_10_ triacylglycerol, mmol/l	0.23 (0.24)	0.21 (0.23)	0.28 (0.22)	0.24 (0.24)	−0.006 (−0.04, 0.03)
Log_10_ alanine aminotransferase	1.61 (0.19)	1.53 (0.18)	1.63 (0.21)	1.53 (0.19)	−0.01 (−0.04, 0.02)
Microalbuminuria, %^a^	20.2% (43/213)	16.1% (35/217)	18.6% (41/220)	17.5% (39/223)	1.22 (0.68, 2.17)^b^
Rose angina questionnaire, % positive	–	12.2% (27/222)	–	12.0% (27/225)	0.98 (0.56, 1.74) ^b^
Michigan neuropathy score	1.91 (1.67)	1.91 (1.88)	1.84 (1.61)	1.78 (1.71)	−0.08 (−0.34, 0.18)
Log_10_ UKPDS 10 year CVD risk^c^	−0.63 (0.21)	−0.68 (0.21)	−0.62 (0.23)	−0.68 (0.22)	−0.02 (−0.04, 0.006)

Values are mean (SD) unless stated

^a^Albumin/creatinine ratio ≥2.5 (men), ≥3.5 (women)

^b^Results expressed as ORs

^c^Excluding *n* = 53 reporting prior CVD

### Functional status, healthy utility, anxiety, well-being, quality of life, treatment satisfaction and relational empathy

Participants in the intervention group reported significantly higher levels of SF-36 physical functioning, SF-36 change in health, health utility (EQ-5D) and satisfaction with diabetes services than those in the comparison group (Table 5). There was no intervention effect on the remaining SF-36 measures, state anxiety, diabetes-specific and general well-being, diabetes-related quality of life, diabetes treatment satisfaction or relational empathy.

**Table 5:** Functional status, health utility, anxiety, quality of life, well-being and satisfaction with treatment at 1 year by study group and between-group differences in the ADDITION-Plus trial

Measure^a^	Comparison group	Intervention group	Difference between groups (95% CI)^b^
SF-36			
Physical functioning	73.3 (27.8)	79.2 (22.5)	5.89 (1.24, 10.85)
Role limitation, physical	71.7 (40.2)	76.0 (35.8)	4.32 (−3.06, 11.29)
Role limitation, emotional	84.4 (32.2)	80.7 (34.2)	−3.69 (−9.98, 1.99)
Social functioning	83.6 (25.3)	85.1 (23.9)	1.51 (−2.93, 6.02)
Mental health	76.3 (19.4)	76.5 (17.3)	0.15 (−3.25, 3.32)
Energy/vitality	56.5 (24.0)	59.4 (20.8)	2.97 (−1.11, 7.21)
Pain	71.6 (27.3)	73.1 (25.2)	1.50 (−3.28, 6.37)
General health perception	59.7 (23.4)	61.2 (20.6)	1.46 (−2.98, 5.51)
Change in health	55.4 (20.9)	64.1 (22.7)	8.72 (4.57, 12.74)
Health utility			
Self-reported general health (1 to 5)	2.94 (0.92)	3.11 (0.87)	0.19 (0.07, 0.31)^c^
EQ-5D (−0.3 to 1.0)	0.81 (0.26)	0.84 (0.20)	0.03 (0.003, 0.06)^c^
Anxiety			
Spielberger State Anxiety (20 to 80)	30.5 (11.0)	29.6 (9.6)	−0.85 (−2.56, 0.85)^c^
Quality of life and well-being			
Quality of life (−9 to 9)	0.96 (1.28)	0.79 (1.05)	−0.17 (−0.38, 0.06)
Diabetes-specific well-being (0 to 36)	28.2 (6.6)	28.8 (6.3)	0.61 (−0.70, 1.76)
General well-being (0 to 36)	26.4 (7.2)	26.9 (6.5)	0.47 (−0.80, 1.81)
Satisfaction with treatment, services and empathy			
Diabetes treatment satisfaction (0 to 36)	30.0 (5.8)	30.6 (5.3)	0.59 (−0.41, 1.72)
General practitioner CARE measure (10 to 50)	39.1 (10.2)	40.3 (9.5)	1.23 (−0.64, 3.17)
Nurse CARE measure (10 to 50)	39.8 (9.8)	40.9 (9.1)	1.14 (−0.78, 2.89)
Satisfaction with diabetes services (1 to 4)	3.43 (0.79)	3.68 (0.56)	0.25 (0.13, 0.38)

Values are mean (SD) unless stated

^a^Values in parentheses represent the possible range for each measure

^b^Model residuals showed non-normality, which could not be resolved after log-transformation. Therefore, 95% CIs were calculated using the semi-parametric bias corrected and accelerated bootstrap regression method (with 1,999 resamples)

^c^Difference between groups is adjusted for baseline values

### Beliefs about behavioural intentions, illness perceptions and habit

Behavioural intentions were higher in the intervention than the comparison group at 1 year, achieving statistical significance for physical activity and medication adherence (Table 6). Illness perceptions, perceived behavioural control and behavioural beliefs were similar in both groups at baseline and 1 year. In addition, there were no differences between groups in the strength of habit and diabetes knowledge levels at 1 year.

**Table 6:** Baseline and follow-up behavioural beliefs, illness perceptions, strength of habit and diabetes knowledge by study group and between-group differences in the ADDITION-Plus trial

Measures^a^	Comparison group		Intervention group		Adjusted difference between groups (95% CI)
	Baseline	1 year	Baseline	1 year	
Intention physical activity (1 to 5)	3.72 (0.83)	3.48 (0.79)	3.78 (0.74)	3.64 (0.81)	0.14 (0.007, 0.27)
Intention diet (1 to 5)	3.71 (0.79)	3.39 (0.84)	3.75 (0.76)	3.51 (0.81)	0.11 (−0.03, 0.25)
Intention medication adherence (1 to 5)	4.45 (0.56)	4.47 (0.60)	4.40 (0.67)	4.57 (0.58)	0.12 (0.01, 0.23)
Intention smoking (1 to 5)	3.16 (1.11)	3.12 (1.06)	3.20 (0.96)	3.20 (1.11)	0.003 (−0.32, 0.33)
Illness perception consequences (1 to 5)	2.90 (0.65)	2.88 (0.69)	2.89 (0.64)	2.95 (0.66)	0.08 (−0.03, 0.18)
Illness perception treatment control (1 to 5)	3.80 (0.52)	3.67 (0.55)	3.75 (0.48)	3.72 (0.52)	0.08 (−0.01, 0.16)
Perceived behavioural control physical activity (1 to 5)	3.72 (0.91)	3.51 (0.90)	3.82 (0.79)	3.67 (0.88)	0.12 (−0.03, 0.26)
Perceived behavioural control diet (1 to 5)	3.75 (0.83)	3.58 (0.85)	3.70 (0.89)	3.67 (0.87)	0.11 (−0.03, 0.26)
Perceived behavioural control medication (1 to 5)	4.49 (0.56)	4.47 (0.56)	4.47 (0.63)	4.56 (0.58)	0.10 (−0.002, 0.20)
Perceived behavioural control smoking (1 to 5)	2.70 (0.97)	2.90 (0.79)	2.79 (1.01)	2.91 (1.07)	−0.07 (−0.44, 0.30)
Behavioural beliefs physical activity (1 to 5)	4.01 (0.75)	3.92 (0.64)	3.98 (0.62)	3.90 (0.72)	−0.002 (−0.11, 0.11)
Behavioural beliefs diet (1 to 5)	3.83 (0.75)	3.75 (0.75)	3.90 (0.68)	3.81 (0.73)	0.02 (−0.10, 0.14)
Perceived effectiveness of lifestyle change (1 to 5)	3.95 (0.72)	3.82 (0.76)	3.91 (0.65)	3.84 (0.71)	0.05 (−0.08, 0.18)
Strength of PA habit change reported at 1 year (1 to 5)	–	3.27 (0.72)	–	3.41 (0.71)	0.14 (−0.05, 0.32)
Strength of diet habit change reported at 1 year (1 to 5)	–	3.66 (0.55)	–	3.64 (0.61)	−0.02 (−0.15, 0.12)
Diabetes knowledge (self-administered questionnaire) (0 to 47)	–	24.8 (9.18)	–	25.8 (8.53)	0.92 (−0.72, 2.57)

Values are mean (SD) unless stated

^a^Values in parentheses represent the possible range for each measure

PA, physical activity

In total, 93% of participants attended the introductory and initial three core-intervention sessions. Intervention group participants reported feeling confident in using the skills they had been taught (mean [SD] score 7.9 [1.7] on a scale of 0 to 10). Self-reported use of skills to increase physical activity and to eat a lower-fat diet was relatively high, ranging from 62% of participants who reported using prompts or reminders to 88% who reported setting achievable goals. Fewer participants reported using these skills to enhance medication adherence, ranging from 50% who reported that they had prepared for setbacks to 68% setting achievable goals. Skills use was lowest among those participants who were trying to stop smoking, with 26% recording or monitoring their progress and 45% reporting that they had set achievable goals. Intervention participants rated their facilitators highly with a mean response of 44 (SD 6.5, on a scale of 10–50).

### Per protocol, subgroup and sensitivity analyses

Per protocol analyses of the primary outcome data replicated our main results, except for the plasma vitamin C level for which the increase over 12 months was slightly greater in the comparison than in the intervention group (*p* = 0.04). Our conclusions were unaffected by the sensitivity analysis for missing data. Similarly, there was no evidence that intervention effects on the primary outcomes differed between participants with screen-detected and recently diagnosed diabetes.

## Discussion

For patients with recently diagnosed diabetes offered intensive treatment in primary care the additional input of a facilitator from outside the practice delivering a theory-based multi-behaviour intervention was not associated with significant improvements in objectively measured health behaviours. There was no difference between trial groups in change from baseline to 1 year in cardiovascular risk factors or self-reported health behaviours. The intervention group reported higher levels of SF-36 physical functioning as well as SF-36 change in health status, health utility and satisfaction with diabetes services at 1 year, and greater changes in diet and activity over the year compared with the comparison group. However, there was no intervention effect on the remaining SF-36 measures, state anxiety, diabetes-specific and general well-being, diabetes-related quality of life, diabetes treatment satisfaction or relational empathy. We found no evidence to support this external behavioural facilitator model of care where the primary care team delivers a well-organised and intensive treatment service.

### Strengths and limitations

The study was carried out in primary care settings where most of the care of individuals with recently diagnosed diabetes takes place in the UK. General practice registers typically cover 99% of all residents living in England [41], and nearly half of the practices approached agreed to take part. Generalisability to more deprived settings with greater ethnic diversity may be limited in light of the non-random recruitment of general practices from a single geographical region.

Internal validity was strong; participants were individually randomised and groups were well matched for baseline characteristics. Participant retention at 1 year was similarly high in both trial groups. We used objective measurement of four key behaviours affecting CVD risk, as well as measuring self-reported behaviour, functional status and well-being. The apparent effect on self-reported change in behaviours over time highlights the need for caution in interpreting the results of trials of behavioural interventions that rely on subjective measures. Clinically important outcomes were measured using standardised equipment and protocols, with trained staff unaware of study group allocation.

The intervention was based on theory and evidence from psychology to support change in behaviour [42], and included a patient-centred approach to facilitating behaviour change, which has been shown to be more effective than didactic interventions in improving CVD risk factors [43, 44]. Quality-assured delivery was enabled by training, ongoing supervision and protocols. The carefully characterised intervention and the objective measurement of health behaviours allowed us to isolate potential effects of the behavioural intervention from other aspects of intensive general practice care.

Plasma drug and cotinine levels were available only for a subset of participants and the timing of blood samples following ingestion of tablets and other factors affecting plasma drug levels were not standardised, which will have reduced the precision of estimates. However, these issues did not differ by study group and while the measure of adherence was less precise, it was less prone to bias than self-report measures. Objective measures of physical activity, smoking and medication adherence at baseline would have improved precision and enabled us to assess change over time. However, such detailed measurement might increase the salience and influence the behaviour of participants in both groups [45]. This is likely to be more relevant to physical activity than to the other health behaviours assessed via blood samples.

### Understanding the results

There may have been limited scope for additional benefit among ADDITION-Plus patients, who were already receiving intensive treatment, including theory-based educational materials and lifestyle advice on all the target behaviours by the primary care team. In addition, there were improvements from baseline in plasma vitamin C levels, self-reported diet and physical activity and cardiovascular risk factors in both study groups, further limiting potential change. The absence of an intervention effect on health behaviours was unlikely to be due to failure to deliver the programme. Attendance at intervention sessions, self-reported use of skills to improve lifestyle behaviours and levels of satisfaction with the programme were all high. Facilitators met monthly to discuss intervention delivery and listened to tape recordings of their sessions. They also received ongoing supervision and support from a clinical psychologist. The intervention was associated with stronger intentions to change physical activity and medication adherence at follow-up. We targeted mediators of behaviour change and included behaviour change techniques from a range of theories. However, the evidence that any of the commonly used theories predict changes in objectively assessed health behaviours is limited. Behaviour change might be enhanced by focusing on fewer behaviours and being more directive in selecting which ones to focus on. Targeting dietary change and physical activity sequentially might be less effective than targeting them simultaneously, but more evidence is needed [46]. Increasing the intensity of the training or of intervention delivery could have enhanced the opportunity to change behaviour, but may not be feasible in routine practice. Facilitator-led care focusing on behaviour change might add value in settings where primary care based intensive treatment is not feasible or effective.

We recorded moderate effects of the intervention on measures of self-reported health status, health utility and physical function. These positive effects occurred independently of any change in objectively measured health behaviours, suggesting a mechanism independent of behaviour change. Other trials have also shown that interventions can improve patient-reported outcomes such as well-being but not behaviour or clinical endpoints [33, 47]. It is not clear whether the improvements were related to the supportive alliances formed with lifestyle facilitators or whether the effect was mediated by a feeling of false reassurance from participating in the intervention [48]. Regardless of the mechanism, self-reported health is an independent predictor of mortality [49]. These improvements warrant further examination, particularly if they persist over the long term when intervention support is withdrawn and can be achieved with a less intensive intervention. Five-year follow-up of the trial cohort is planned.

Recent attempts to promote behaviour change in individuals soon after the diagnosis of diabetes have had mixed effects. In the Diabetes Education and Self-Management for Ongoing and Newly Diagnosed Diabetes (DESMOND) trial (ISRCTN17844016), 824 newly diagnosed patients were cluster randomised to receive either a 6 h structured group-education programme focused on behaviour change or usual care (comparison group) [50]. The DESMOND trial participants had a mean age of 60 years, included more men than women (55%), had a mean BMI of approximately 32 kg/m^2^ and were, therefore, broadly similar to ADDITION-Plus participants. The intervention was based on similar theories of learning used in the ADDITION-Plus trial and focused on lifestyle factors such as food choice and physical activity. Compared with the control group, the intervention group achieved greater improvements in weight loss and smoking cessation and positive improvements in belief about illness, but no difference in HbA_1c_ levels after 1 year. Self-reported physical activity levels were higher in the intervention than in the comparison group at all time points, but were not significantly different at 1 year. There was no objective measurement of behaviour change in this trial cohort and it was difficult to elucidate exactly which components of the intervention were associated with the observed improvements. Further, the DESMOND control group was different from our comparison group, where individuals were already receiving intensive treatment for diabetes.

In the Early ACTID trial, 593 recently diagnosed patients with a mean age of 60 years were randomised to receive usual care (control group), an intensive diet intervention (6.5 h of individual counselling by a dietitian/nurse over 1 year) or an intensive diet intervention plus a pedometer-based activity programme [7]. After 12 months, there were significant improvements in glycaemic control, insulin resistance and body weight in both intervention groups compared with the control group; however, the addition of the activity intervention conferred no extra benefit. Accelerometry data indicated that individuals in the diet and activity group increased their physical activity significantly more than those in the other two groups. However, the lack of measurement of dietary change or any other health behaviour again makes it difficult to explain the beneficial effects observed in this trial. Other studies examining behavioural change in type 2 diabetes patients tend to be small, of shorter duration and to focus on individuals later in the disease trajectory. No published trial to date captures objective measurement of four key health behaviours in recently diagnosed patients.

## Conclusion

A facilitator-led, individually tailored multiple behaviour change intervention over 1 year did not improve objectively measured health behaviours or cardiovascular risk factors among people with recently diagnosed type 2 diabetes receiving intensive treatment in UK primary care. Effects on self-reported changes in diet and physical activity were not confirmed by objective data, highlighting the need for caution in interpreting trials of behavioural interventions that rely on subjective measures. Enabling primary care teams to deliver a well-organised intensive treatment service to newly diagnosed patients should remain a priority for policymakers and commissioners.

## Electronic supplementary material

Below is the link to the electronic supplementary material.

ESM (PDF 88 kb)

## Abbreviations

ADDITION: Anglo–Danish–Dutch Study of Intensive Treatment in People with Screen Detected Diabetes in Primary Care
CARE: Consultation and Relational Empathy
CVD: Cardiovascular disease
DESMOND: Diabetes Education and Self-Management for Ongoing and Newly Diagnosed Diabetes
Early ACTID: Early Activity in Diabetes
EQ-5D: EuroQol 5-D
Look-AHEAD: Action for Health Diabetes
MARS: Medication Adherence Report Schedule
PAEE: Physical activity energy expenditure
SF-36: Short-Form 36
TPB: Theory of planned behaviour
UKPDS: UK Prospective Diabetes Study
