# Supplementary material for: Multiple behaviour change intervention and outcomes in recently diagnosed type 2 diabetes: the ADDITION-Plus randomised controlled trial
Source: Diabetologia. 2014 Apr 24;57(7):1308–19. doi: 10.1007/s00125-014-3236-6 (PMC4052009; doi:10.1007/s00125-014-3236-6)
Supplement: Supplementary file 1 — (PDF 88 kb) [file 125_2014_3236_MOESM1_ESM.pdf]

Electronic Supplementary Material

Multiple behaviour change intervention and outcomes in recently diagnosed type 2 diabetes: the *ADDITION-Plus* randomised controlled trial (clinical trials registration number ISRCTN99175498)

Methods

Intervention

Comparison group: Intensive treatment

A number of features were added to routine multi-disciplinary primary care of diabetes to achieve intensive treatment in both trial groups as previously described [1, 2]. These included practice-based educational meetings, treatment algorithms, target setting, audit and feedback, individual-level, diabetes education delivered by practice nurses (supported by theory-based written materials [3]). The intensive treatment algorithms included lifestyle advice and recommended stepwise target-led drug treatment to reduce hyperglycaemia, blood pressure, hyperlipidaemia and microalbuminuria, as well as prescription of aspirin to those without contraindications.

Intervention group: Intensive treatment plus facilitator-led behaviour change intervention

Participants in this group received intensive treatment (described above) plus a facilitator-led, individually tailored behaviour change intervention, based on psychological theory and evidence. Full details of the intervention have been described previously [2]. The intervention was delivered by three female trained lifestyle facilitators, who were not part of the general practice team. Two had a professional background in nursing and one in social work. Initial and refresher training took seven days and covered the delivery of each session, including the theoretical basis and practice in the behaviour change techniques. These were supplemented with training about the target behaviours (dietary change, smoking cessation etc.), and observation in pilot practices. The facilitators used detailed protocols to guide each contact with the participant and received on-going supervision and feedback from a clinical psychologist, informed by assessment of tape-recorded consultations, enabling tight quality assurance of intervention delivery. The intervention was designed to build on the diabetes education delivered by practice nurses and intensive treatment by the practice team. The behaviours targeted in the intervention were physical activity, dietary intake, medication adherence, and smoking cessation. Hypothesised mediators of behaviour change targeted in the intervention included illness perceptions based on Leventhal’s Common-Sense Model [3] and beliefs about behaviour change based on the Theory of Planned Behaviour (TPB) [4]. The intervention targeted instrumental (e.g. health) and affective beliefs about changing specific behaviours, (lack of) encouragement by important others (subjective norm), and perceived barriers to and facilitators of

behaviour change (perceived behavioural control). Facilitators taught patients a range of self-regulatory skills to achieve behaviour change and maintenance over time which was supported by a manual describing the skills. The facilitators used behaviour change techniques which were specified in the protocols for each contact and could be mapped onto the TPB (e.g., giving information, strengthening motivation), Operant Theory (identifying cues for action and reinforcement of behaviour change or effort), Carver and Scheier's Control Theory (e.g., goal setting, action planning, self-monitoring) and Relapse Prevention Theory (e.g., preparing for and dealing with setbacks) [4-7]. Each patient initially selected to work on the behaviour(s) they most wanted to change and felt most confident about changing. The facilitator later discussed with them other domains in which change was possible and encouraged patients to consider setting additional goals. Interested patients were offered a pedometer to self-monitor their daily physical activity. The intervention was delivered over one year at the participants' surgeries. It included a one-hour introductory meeting followed by six 30-minute meetings and four brief phone calls. Facilitators visited participant's homes or workplaces if it was not possible to meet in the surgery.

Secondary outcomes

Biochemical measures: HbA_{1c} was analysed in venous samples at baseline and at follow-up by ion-exchange high-performance liquid chromatography on a Tosoh machine (Tosoh Bioscience, Redditch, UK). Serum total cholesterol, HDL-cholesterol and triglycerides were measured by means of enzymatic techniques (Dade Behring Dimension analyser, Newark, USA). Plasma creatinine was analysed with kinetic colorimetric methods, and urine albumin by rate nephelemetry (Dade Behring Nephelometer II, Newark, USA). Plasma levels of urea and electrolytes, bilirubin, alanine aminotransferase (ALT), aspartate aminotransferase (AST), alkaline phosphatase, and thyroid stimulating hormone (TSH) and urine levels of creatinine were assayed by means of the Dade Behring Dimension analyser. The albumin-to-creatinine ratio (ACR) was measured on a random spot urine sample.

[1] Echouffo-Tcheugui JB, Simmons RK, Williams KM, et al. (2009) The ADDITION-Cambridge trial protocol - a cluster randomised controlled trial of screening for type 2 diabetes and intensive treatment for screen-detected patients. BMC Public Health 9: 136

[2] Griffin SJ, Simmons RK, Williams KM, et al. (2011) Protocol for the ADDITION-Plus study: a randomised controlled trial of an individually-tailored behaviour change intervention among people with recently diagnosed type 2 diabetes under intensive UK general practice care. BMC Public Health 11: 211

[3] Leventhal H, Brissette, I., Leventhal, E.A. (2003) The common-sense model of self-regulation of health and illness. In: Cameron LD, Leventhal, H. (ed) The Self-Regulation of Health and Illness Behaviour. Routledge, London, pp 42-65

[4] Ajzen I (1991) The Theory of Planned Behavior. Organizational Behavior and Human Decision Processes 50: 179-211

[5] Carver CS, Scheier, M.F. (1999) Themes and issues in the self-regulation of behavior. In: Wyer RS (ed) Perspectives on behavioral self-regulation. Advances in Social Cognition, Volume XII. Lawrence Erlbaum Associates, London

[6] Kazdin AE (2001) Behavior Modification in Applied Settings. Wadsworth, Belmont, CA

[7] Marlatt GA, Gordon, J.R. (1985) Relapse prevention: Maintenance strategies in the treatment of addictive behaviors. Guildford Press, New York
